# The impact of COVID-19 pandemic on headache symptoms and drug withdrawal among patients with medication overuse headache: a cross-sectional study

**DOI:** 10.1186/s10194-021-01256-0

**Published:** 2021-05-21

**Authors:** Changling Li, Yanbo Li, Mengmeng Ma, Yang Zhang, Jiajia Bao, Wenjing Ge, Yanqin Liu, Cheng Peng, Li He

**Affiliations:** Department of Neurology, West China Hospital of Sichuan University, No. 37 Wainan Guoxue Xiang, Chengdu, 610041 China

**Keywords:** COVID-19, Pandemic, Medication overuse headache, Prophylactic, Withdrawal

## Abstract

**Background:**

Coronavirus disease 2019 (COVID-19) bring about a range of psychological distress and symptom deterioration to headache patients especially to some migraineurs. Compared to migraineurs or normal control, medication overuse headache (MOH) patients are more likely to experience a worse psychological distress and poorer outcome in non-COVID-19 time. However, in COVID-19 pandemic, whether MOH patients would have greater physical and mental symptom deterioration or worse relief of headache symptoms and medications overuse remained unclear. We aim to investigate the impact of COVID-19 on MOH patients to guide for a better management in this study.

**Methods:**

We enrolled MOH patients who were diagnosed and treated at headache clinic of West China Hospital. Information of the pre-pandemic 3 months period and COVID-19 pandemic period was collected. Univariate and multivariate logistic regression were performed to identify independent factors associated with changes in headache symptoms and drug withdrawal.

**Results:**

Seventy-eight MOH patients were enrolled into the study ultimately. In comparison to pre-pandemic period, fewer MOH patients reported decreased headache days, intensity and days with acute medications per month during the pandemic. Available access to regular prophylactic medications was significantly associated with a reduction of at least 50% in headache days and decrease in headache intensity per month with respective odds ratios of 39.19 (95% CI 3.75–409.15, *P* = 0.002) and 10.13 (95% CI 2.33–44.12, P = 0.002). Following abrupt withdrawal and high educational level were both significant factors in decreasing headache intensity. Male sex was significantly associated with decrease in days with acute medication per month during the pandemic (odds ratios 4.78, 95%CI 1.44–15.87, *P* = 0.011).

**Conclusions:**

Our findings reflect that MOH patients experienced a worse relief of headache symptoms and drug withdrawal during the pandemic. Available access to regular prophylactic medications was the significant independent factor for improvement of headache symptoms. Male sex was significantly associated with decreased days with acute medications per month.

## Introduction

Coronavirus disease 2019 (COVID-19) caused by severe acute respiratory syndrome coronavirus 2 (SARS-CoV-2) [1] has already rapidly spread around the world as a pandemic after its first report in Wuhan, China on December 12th 2019 [2–5]. As of December 27th 2020, there were more than 79.2 million confirmed cases and more than 1.7 million deaths caused by COVID-19 worldwide [6]. The outburst of high contagious and deadly COVID-19 lead to a period of protective equipment insufficiency and prompted the execution of the policies of home quarantine and social isolation. These are all considered to bring about a range of psychological distress among the public [7]. For patients with chronic neurological diseases, such as multiple sclerosis, neurodegenerative disease, chronic headache and etc., they were considered to probably experience psychological distress such as despair or depression under the impact of the COVID-19 pandemic as Bhaskar stated, which resulted in non-compliance, potential relapse and worsening of the condition [8].

Medication overuse headache (MOH) is an important secondary chronic headache disorder which gets little attention. It is caused by long-term regular overuse of analgesics or acute medications in patients suffering from primary headache disorders such as migraine and tension-type headache (TTH) or other secondary headaches [9–11]. Globally, MOH makes an influence on approximately 60 million people, ranking one of the global top 20 causes of disability [12] and resulting in significant socioeconomic consequences [13–16]. It is a troublesome disease that requires more medical care and regular follow-up because of its relatively high relapse rate [10].

In non-COVID-19 time, MOH patients are more likely to comorbid with psychological distress (including depression and anxiety) than normal population and migraineurs, and the psychological distress is tightly associated with poor outcomes of withdrawal therapy among MOH patients [9, 17–22].

Whereas, after COVID-19 burst out, migraineurs were reported to have had suffered increased migraine frequency and pain intensity as well as overuse of analgesics and acute migraine treatments, due to high levels of psychosocial distress and COVID-19-related concerns [23, 24]. Thus, like the phenomenon we observe under non-COVID-19 circumstances, we considered that MOH patients might be inclined to experience a greater symptom deterioration or worse relief of the condition than migraineurs did under the impact of the COVID-19 pandemic. However, what MOH patients actually suffered during the pandemic remained unclear.

Thus, in this cross-sectional study, we aimed to investigate the change and relevant factors of headache days, headache intensity, and days with acute medication per month in MOH patients during the COVID-19 pandemic, compared by those in pre-pandemic period, to provide guides for the management of MOH patients during the time attacked by this public health emergency.

## Methods and materials

This cross-sectional study was conducted in forms of telephone surveys on MOH patients who were recruited from the headache database between April and July, 2020. We used a designed questionnaire containing several validated scales to obtain the information about headache symptoms, psychosocial status and therapeutic condition of the MOH group in its pre-pandemic 3 months period and COVID-19 pandemic period. This telephone-based questionnaire ended in July, 2020.

### Participants

We recruited patients who were diagnosed with MOH according to the International Classification of Headache Disorders, 3rd edition (ICHD-III) criteria [25] from a tertiary headache clinic of West China Hospital. Patients were identified from an existing database with the age beyond 18 years old. Routinely, they were followed up monthly by a neurology specialist once they were incorporated into our database. The patients we followed were receiving MOH treatments, and some had been overuse-free while others had stayed overused in pre-pandemic 3 months period. Patients were excluded if they were confirmed COVID-19-positive patient; or they had a history of psychiatric disorders; or they were unwilling to receive telephone interview or unable to complete the questionnaires.

The study was reviewed by the Ethics Committee of West China Hospital, Sichuan University, and verbal informed consent was provided by all participants.

### Data collection

The telephone-based study was conducted by two neurologists who did not know the purpose of the study. The following items were included in the questionnaire:

(1) Demographic variables (sex, age, marital status, living with others, educational level, and household income monthly). (2) MOH related information of pre-pandemic 3 months period and COVID-19 pandemic period (preexisting headache, duration of medication overuse, available access to regular prophylactic medications, headache days per month, headache intensity [Visual Analogue Scale score with range of 0–10] per month, days per month with acute medication, and Migraine Disability Assessment Scale [MIDAS] score). (3) COVID-19 related information (epidemiological area exposure history, suspected COVID-19 cases themselves or in their relatives, COVID-19 cases in respondent’s residential community, feel of difficulty in controlling emotions, frequency of COVID-19 related dreams, duration per day spent on following news of COVID-19 pandemic, level of concern about the pandemic [4 points scales from “relatively unconcerned or very unconcerned” to “very concerned”], psychological disorder history, psychological distress during the COVID-19 pandemic [Mandarin version of Kessler 6-item psychological distress scale (K-6 scale) [26]], and COVID-19 related stressor investigation scale).

Psychological distress during the COVID-19 pandemic was measured by using K-6 scale, which was designed by Kessler et al. It was frequently used in large-scale surveys to assess non-specific psychological distress over the past month [27, 28]. The scale includes 6 items: “nervous”, “hopeless”, “restless or fidgety”, “so depressed that nothing could cheer you up”, “everything is an effort” and “worthless” [27]. The Link 5 score is applied for each item, “0” means none of the time, “1” means a little of the time, “2” means some of the time, “3” means most of the time, and “4” means all the time [29]. K-6 total score >  12 was considered a severe psychological stress and the mandarin version has been proven to have good reliability and validity [26].

COVID-19 related stressor was assessed by using a self-designed stressor scale based on the study of Ke-rang Zhang et al. during the pandemic of SARS (Severe Acute Respiratory Syndrome). The scale was divided into 7 aspects, including 18 items: (1) Fear for getting COVID-19 (4 items): including yourself, family members, friends and colleagues. (2) Physical and psychological distress caused by isolation (2 items). (3) Inconvenience caused by work and life changes during COVID-19 pandemic (2 items). (4) Social discrimination (2 items): including subjective feelings and objective facts. (5) Limited social function (4 items): including school enrollment, employment, making friends, wedding, interpersonal communication and other aspects. (6) Economic losses (2 items): temporary and long-term economic losses. (7) Coronavirus death of family members or friends (2 items). The Link 5 score is performed for each item, “1” means completely inconsistent, “2” means less inconsistent, “3” means general, “4” means more consistent, and “5” means completely consistent. The score of 2 for each item indicates that patients were mildly affected in this item. The higher the score, the more severe the response to stress induced by COVID-19 pandemic was.

In this study, the main outcomes were set as changes in headache days, headache intensity and days with acute medication per month from 3 months pre-pandemic to COVID-19 pandemic. We used reduction of at least 50% to illustrate the change in headache days per month, and reduction of at least 50% in headache days per month was defined as improvement in headache days among MOH patients [30]. In addition, relevant factors were also analyzed for these changes among MOH patients.

### Statistical analysis

Statistical Product and Service Solutions (SPSS) Software 22.0 (SPSS Inc., Chicago, IL, USA) for Windows was used for data analysis. Continuous variables were represented by means and standard deviation or median and percentile, and categorical variables were represented by absolute numbers and percentages. Univariate logistic regression was performed to determine the association between the clinical variables and change in headache days, headache intensity and days with acute medication per month. Multivariate logistic regression was used to explore factors which were independently associated with the change. Variables associated with *P* < 0.10 in the univariate analysis and covariates of interest in previous articles were incorporated in the multivariate regression model by using the enter method.

All tests were two-sided and *P* < 0.05 was considered statistically significant.

## Results

One hundred and ten patients with MOH were invited to participate in the questionnaire survey, 26 patients rejected the interview or were unable to complete the questionnaires; 6 patients were excluded because they had mental disease currently or before. In the end, 78 MOH patients (70.9%) were enrolled.

### Demographic and clinical characteristics

Among all the 78 enrolled patients, the mean age was 51.6 (±10.5) years, and a total of 28 (35.9%) patients with MOH were male sex. The majority of the patients were married (93.6%) and living with others (84.6%). Lower educational level and lower monthly household income were reported by most patients (Table 1). Besides, episodic migraine and TTH were present in 50% (39/78) of the preexisting headache diagnoses, and the median duration of medication overuse was 3.0 (2.0–7.3) years. Available access to regular prophylactic medications only occurred in 41.0% (32/78) patients during the pandemic. Less than 50% patients followed abrupt withdrawal. The MIDAS score was low during the pandemic with the median score of 0.0 (0.0–7.0).

**Table 1:** Distribution of different variables in MOH patients

Characteristic	MOH patients (*n* = 78) *
Sex, male	28 (35.9%)
Age, year	51.6 ± 10.5
Marital status, married	73 (93.6%)
Living with others	66 (84.6%)
Education level, year	
≤ 12	64 (82.1%)
> 12	14 (17.9%)
Household income monthly ^a^, RMB	
0–4999	41 (52.5%)
5000–9999	22 (28.2%)
10,000–14,999	8 (10.3%)
15,000–19,999	3 (3.9%)
> 20,000	4 (5.1%)
Preexisting headache diagnoses	
Chronic migraine	5 (6.4%)
Episodic migraine and TTH	39 (50.0%)
Chronic TTH	34 (43.6%)
Duration of medication overuse, year	3.0 (2.0–7.3)
Available access to regular prophylactic medications	32 (41.0%)
Following abrupt withdrawal	34 (43.6%)
Headache days per month in the pre-pandemic 3 months period	10.0 (3.0–18.5)
Headache intensity per month in the pre-pandemic 3 months period	5.0 ± 2.3
Days per month with acute medication in the pre-pandemic 3 months period	10.0 (1.0–15.0)
MIDAS score during the COVID-19 pandemic	0.0 (0.0–7.0)
History of epidemiological area exposure	2 (2.6%)
Suspected COVID-19 cases themselves or in their relatives	1 (1.3%)
COVID-19 cases in respondent’s residential community	2 (2.6%)
Level of concern about COVID-19 pandemic	
Very concerned	4 (5.1%)
Concerned	22 (28.2%)
Average	11 (14.1%)
Relatively unconcerned or very unconcerned	41 (52.6%)
Duration spent on following news of pandemic, hours per day	0.6 ± 1.1
Feel of difficulty in controlling emotion during the COVID-19 pandemic	6 (7.7%)
Frequency of COVID-19 related dreams	
Most of time or sometimes	0
None	78 (100.0%)
Stressors total score during the COVID-19 pandemic	30.8 ± 10.0
Fear for getting COVID-19 (4 items)	6.9 ± 3.1
Physical and psychological distress caused by isolation (2 items)	3.0 ± 1.9
Inconvenience caused by work and life changes (2 items)	3.7 ± 2.6
Social discrimination (2 items)	2.1 ± 0.6
Limited social function (4 items)	8.9 ± 4.3
Economic losses (2 items)	4.2 ± 2.5
Coronavirus death of family members or friends (2 items)	2.0 ± 0.1
Kessler 6-item psychological distress scale during the COVID-19 pandemic	1.0 (0.0–3.0)
Nervous	0.0 (0.0–1.0)
Hopeless	0.0 (0.0–0.0)
Restless or fidgety	0.0 (0.0–1.0)
So depressed that nothing could cheer you up	0.0 (0.0–1.0)
Everything is an effort	0.0 (0.0–0.0)
Worthless	0.0 (0.0–0.0)
Severe psychological distress	0

*Values are n (%), mean ± SD, or median (quartiles)

^a^: 1 RMB = 0.141 USD on 30 June 2020

### COVID-19 related information

Less than 3% of the patients reported previous epidemiological area exposure or recent community exposure to COVID-19 cases (eg. sharing an environment with COVID-19 cases in the same residential community). One of the patients was ever considered to be suspected COVID-19 patient, however, he was ultimately confirmed to be COVID-19-free before our survey. Few patients had difficulty in controlling emotion and no one had COVID-19 related dreams. The duration spent on following COVID-19 pandemic news was also low with mean of 0.6 (±1.1) hours per day, and more than half of the patients were relatively unconcerned or very unconcerned about the pandemic. In addition, on basis of the stressor scale, MOH patients were found to be affected, to a minor degree, by multiple COVID-19 related aspects excluding the item of COVID-19 death. The mean score of the overall stressors was 30.8 (±10.0) (shown in Table 1). Psychological distress evaluated by K-6 was low in all the patients with the median score of 1.0 (0.0–3.0) and no one experienced severe psychological distress.

### Main outcomes

Compared to pre-pandemic period, headache days per month unchanged in more than half of patients (53.8%), and increased in 16.7% (13/78) of patients during the pandemic. Only 20.5% (16/78) of the patients reported a reduction of 50% or more in headache days per month (Table 2).

**Table 2:** Outcome changes during COVID-19 pandemic in MOH patients

Outcome	MOH patients^a^
Change in headache days per month	
Increase	13 (16.7%)
Unchanged	42 (53.8%)
Decrease	23 (29.5%)
50% reduction	16 (20.5%)
Change in headache intensity per month	
Increase	8 (10.3%)
Unchanged	43 (55.1%)
Decrease	27 (34.6%)
Change in days per month with acute medication	
Increase	11 (14.1%)
Unchanged	46 (59.0%)
Decrease	21 (26.9%)
Change from medication overuse to non-overuse	9 (11.5%)

^a^Values are n (%), mean ± SD

With regard to the headache intensity per month, 8 (10.3%) patients reported increased headache intensity and 43 (55.1%) patients remained unchanged during the pandemic. Left 27 (34.6%) patients experienced decreased headache intensity per month (Table 2).

Similar changes were observed in the outcome of days per month with acute medication (Table 2). During the pandemic, 71.0% (22/31) of the original overusers still overused acute medications and 29.0% of them were overuse-free. Meanwhile, 89.4% (42/47) of the original non-overusers remained overuse-free and 10.6% of them overused acute medications again (*P* < 0.001).

### Assessment of the relevant factors associated with a reduction of at least 50% in headache days per month

The univariate logistic analysis showed that available access to regular prophylactic medications and following abrupt withdrawal were significantly associated with the reduction of at least 50% in headache days per month (*P* < 0.05, Table 3). Multivariate logistic regression showed that available access to regular prophylactic medications was an independent predictor for a reduction of at least 50% in headache days per month with OR of 39.19 during the COVID-19 pandemic (95% CI 3.75–409.15, *P* = 0.002; Table 3).

**Table 3:** Univariate and multivariate logistic analysis to identify independent variables reducing headache days (≥ 50%)

Variables	Univariate		Multivariate	
	OR(95%CI)	P	OR(95%CI)	P
Sex, male	2.1 (0.69–6.41)	0.192	–	–
Age, year	1.03 (0.97–1.09)	0.329	–	–
Marital status, married	1.03 (0.11–9.95)	0.997	–	–
Education level (> 12 years)	1.07 (0.26–4.40)	0.925	–	–
Household income monthly	1.14 (0.38–3.42)	0.818	–	–
Preexisting headache diagnoses (CTTH vs. others)	0.73 (0.24–2.25)	0.582	0.50 (0.09–2.82)	0.434
Duration of medication overuse	0.90 (0.76–1.05)	0.182	–	–
Available access to regular prophylactic medication	39.71 (4.86–324.16)	0.001^*^	39.19 (3.75–409.15)	0.002^*^
Following abrupt withdrawal	5.46 (1.57–18.95)	0.008^*^	3.47 (0.65–18.59)	0.147
Headache days per month in the pre-pandemic 3 months period	1.00 (0.95–1.05)	0.953	1.06 (0.91–1.22)	0.469
Days per month with acute medication in the pre-pandemic 3 months period	0.98 (0.93–1.03)	0.448	0.96 (0.84–1.10)	0.532
History of epidemiological area exposure	0.25 (0.02–4.16)	0.331	–	–
Suspected COVID-19 cases themselves or in their relatives	–	1.000	–	–
COVID-19 cases in respondent’s residential community	0.25 (0.02–4.16)	0.331	–	–
Level of concern about COVID-19 pandemic	1.56 (0.52–4.72)	0.430	–	–
Duration spent on following news of pandemic	1.65 (0.91–2.98)	0.099	1.23 (0.65–2.32)	0.527
Feel of difficulty in controlling emotion during the COVID-19 pandemic	2.07 (0.34–12.49)	0.426	–	–
Stressors total score during the COVID-19 pandemic	1.02 (0.97–1.08)	0.409	–	–
Fear for getting COVID-19	0.96 (0.80–1.16)	0.680	–	–
Physical and psychological distress caused by isolation	1.13 (0.86–1.47)	0.390	1.08 (0.69–1.70)	0.731
Inconvenience caused by work and life changes	1.02 (0.83–1.25)	0.870	1.18 (0.83–1.68)	0.355
Social discrimination	–	0.999	–	–
Limited social function	1.07 (0.94–1.21)	0.305	–	–
Economic losses	1.17 (0.95–1.45)	0.144	–	–
Coronavirus death of family members or friends	–	1.000	–	–
Kessler 6-item psychological distress scale during the COVID-19 pandemic	0.89 (0.69–1.15)	0.356	–	–

*CI* Confidence interval; *: Statistically significant at *P* < 0.05

### Assessment of the relevant factors associated with decrease in headache intensity per month

Available access to regular prophylactic medications and following abrupt withdrawal were significantly associated with decrease in headache intensity per month in the univariate logistic regression (*P* < 0.05, Table 4). Available access to regular prophylactic medications (OR 10.13, 95% CI 2.33–44.12, *P* = 0.002), following abrupt withdrawal (OR 5.12, 95% CI 1.27–20.54, *P* = 0.021) and high educational level (OR 5.98, 95% CI 1.19–30.06, *P* = 0.030) were significantly associated with decrease in headache intensity per month when potential factors were incorporated into the multivariate logistic regression model (Table 4).

**Table 4:** Univariate and multivariate logistic analysis to identify independent variables decreasing headache intensity per month

Variables	Univariate		Multivariate	
	OR(95%CI)	P	OR(95%CI)	P
Sex, male	1.08 (0.41–2.84)	0.879	–	–
Age, year	1.00 (0.96–1.05)	0.965	–	–
Marital status, married	0.33 (0.05–2.09)	0.237	–	–
Education level (> 12 years)	3.16 (0.96–10.34)	0.058	5.98 (1.19–30.06)	0.030^*^
Preexisting headache diagnoses (CTTH vs. others)	1.06 (0.41–2.70)	0.912	2.05 (0.49–8.50)	0.323
Duration of medication overuse	0.87 (0.75–1.00)	0.051	0.90 (0.76–1.06)	0.192
Available access to regular prophylactic medication	5.29 (1.93–14.50)	0.001^*^	10.13 (2.33–44.12)	0.002^*^
Following abrupt withdrawal	4.38 (1.62–11.83)	0.004^*^	5.12 (1.27–20.54)	0.021^*^
Headache intensity per month in the pre-pandemic 3 months period	1.11 (0.90–1.37)	0.314	1.12 (0.84–1.50)	0.449
History of epidemiological area exposure	–	0.999	–	–
Suspected COVID-19 cases themselves or in their relatives	–	1.000	–	–
COVID-19 cases in respondent’s residential community	0.52 (0.03–8.66)	0.649	–	–
Level of concern about COVID-19 pandemic	2.08 (0.81–5.37)	0.131	–	–
Duration spent on following news of pandemic, hours per day	1.50 (0.86–2.63)	0.154	–	–
Feel of difficulty in controlling emotion during the COVID-19 pandemic	0.94 (0.16–5.49)	0.945	–	–
Stressors total score during the COVID-19 pandemic	1.02 (0.97–1.07)	0.393	–	–
Fear for getting COVID-19	1.01 (0.87–1.17)	0.913	–	–
Physical and psychological distress caused by isolation	0.99 (0.77–1.28)	0.965	0.78 (0.54–1.12)	0.175
Inconvenience caused by work and life changes	1.12 (0.94–1.33)	0.198	1.32 (1.00–1.73)	0.051
Social discrimination	1.14 (0.54–2.41)	0.727	–	–
Limited social function	1.03 (0.92–1.15)	0.584	–	–
Economic losses	1.10 (0.91–1.32)	0.332	–	–
Coronavirus death of family members or friends	–	1.000	–	–
Kessler 6-item psychological distress scale during the COVID-19 pandemic	0.96 (0.80–1.16)	0.672	–	–

*CI* Confidence interval; *: Statistically significant at *P* < 0.05

### Assessment of the relevant factors associated with decrease in days per month with acute medication

Male sex was significantly associated with the decrease in days per month with acute medication in univariate logistic analysis (*P* = 0.021, Table 5). Multivariate logistic regression showed that male sex was a significant independent factor for the decrease in days per month with acute medication with OR of 4.78 (95% CI 1.44–15.87, *P* = 0.011; Table 5).

**Table 5:** Univariate and multivariate logistic analysis to identify independent variables decreasing days with acute medication

Variables	Univariate		Multivariate	
	OR(95%CI)	P	OR(95%CI)	P
Sex, male	3.42 (1.21–9.67)	0.021^*^	4.78 (1.44–15.87)	0.011^*^
Age, year	1.01 (0.97–1.06)	0.581	–	–
Marital status, married	1.51 (0.16–14.33)	0.720	–	–
Education level (> 12 years)	1.11 (0.31–4.00)	0.878	–	–
Household income monthly	1.71 (0.62–4.69)	0.300	–	–
Preexisting headache diagnoses (CTTH vs. others)	1.25 (0.46–3.42)	0.663	–	–
Duration of medication overuse	0.96 (0.87–1.05)	0.336	0.96 (0.88–1.05)	0.359
Available access to regular prophylactic medication	2.47 (0.89–6.85)	0.083	2.31 (0.63–8.43)	0.206
Following abrupt withdrawal	1.63 (0.59–4.45)	0.344	2.82 (0.67–11.91)	0.160
Headache intensity per month in the pre-pandemic 3 months period	1.07 (0.86–1.34)	0.535	1.04 (0.79–1.38)	0.780
Days per month with acute medication in the pre-pandemic 3 months period	1.04 (0.99–1.08)	0.127	1.05 (0.99–1.11)	0.131
History of epidemiological area exposure	0.36 (0.02–5.98)	0.474	–	–
Suspected COVID-19 cases themselves or in their relatives	–	1.000	–	–
COVID-19 cases in respondent’s residential community	0.36 (0.02–5.98)	0.474	–	–
Level of concern about COVID-19 pandemic	0.68 (0.42–1.12)	0.132	–	–
Duration spent on following news of pandemic, hours per day	1.10 (0.71–1.71)	0.656	–	–
Feel of difficulty in controlling emotion during the COVID-19 pandemic	1.40 (0.24–8.24)	0.714	–	–
Stressors total score during the COVID-19 pandemic	1.00 (0.96–1.06)	0.866	–	–
Fear for getting COVID-19	0.94 (0.80–1.12)	0.505	–	–
Physical and psychological distress caused by isolation	0.98 (0.74–1.28)	0.863	0.93 (0.67–1.29)	0.664
Inconvenience caused by work and life changes	1.00 (0.83–1.21)	0.979	1.10 (0.87–1.39)	0.426
Social discrimination	–	0.999	–	–
Limited social function	1.05 (0.93–1.18)	0.430	–	–
Economic losses	1.06 (0.87–1.29)	0.591	–	–
Coronavirus death of family members or friends	–	1.000	–	–
Kessler 6-item psychological distress scale during the COVID-19 pandemic	1.07 (0.89–1.29)	0.497	–	–

*CI* Confidence interval; *: Statistically significant at *P* < 0.05

## Discussion

MOH is often overlooked despite its heavy disease burden, and currently it is more widely recognized as a separate diagnostic entity secondary to a pre-existing headache disorder in the ICHD-III [11]. Since MOH patients are more likely to suffer from psychological distress and easy to have bad outcome, headache specialists need to pay more attention to them, especially during the COVID-19 pandemic. We conducted this study to investigate the change in headache days, headache intensity, days with acute medication per month and their relevant factors among MOH patients from 3 months pre-pandemic to COVID-19 pandemic for a better management. Few MOH patients in pandemic period had decreased headache intensity, decreased days with acute medications, and a reduction of at least 50% in headache days per month. We found that available access to regular prophylactic medications was significantly associated with improvement of headache symptoms. Meanwhile, higher educational level and following abrupt withdrawal were significant independent factors for decreased headache intensity per month. Male sex was significantly associated with decrease in days with acute medication per month.

During the pandemic, we found few MOH patients experiencing improved headache days and intensity compared with pre-pandemic period. These results differ from the findings of several published studies conducted in MOH patients under non-COVID-19 condition [14, 17, 31, 32], which found significant decrease in headache days and intensity per month in most MOH patients with regular prophylactic medications. The inconsistency is probably due mainly to the difficulty of accessing to regular prophylactic medications during the pandemic on basis of our analysis. In the present study, only 41.0% of MOH patients could obtain regular prophylactic medications during the pandemic, while in the previous studies the majority of MOH patients could receive regular and timely prophylactic medications. On one hand, all the resources became strained and supplements and medical services were reconfigured to meet the demands of fighting for COVID-19 in the midst of pandemic. About half of the patients with chronic diseases were unable to receive regular medications during the pandemic as the World Health Organization stated from a survey conducted in 155 countries [33]. On the other hand, the social distancing and the lockdown have critically affected the ongoing care, access and support of outpatients [7]. Especially for chronic headache [8] including MOH patients, stress is increasing on giving them the regular medical care and medications in the drearier light of COVID-19.

Available access to regular prophylactic medications was significantly associated with the reduction of at least 50% in headache days and decreased headache intensity per month during the pandemic. The finding was consistent with the previous study of Diener et al. who found that receiving regular prophylactic medications could lead to a reduction in headache days and headache intensity among MOH patients in non-COVID-19 time [9]. Besides, following abrupt withdrawal was also the independent factor for decreased headache intensity in our study. The results showed that as the fact in non-COVID-19 time [14, 31, 34] combined therapy of abrupt withdrawal with prophylactic medications could also decrease headache days and headache intensity per month in MOH patients during the COVID-19 pandemic. As a series of current guidelines and authors recommended, receiving withdrawal with simultaneous prophylactic medications is the most effective treatment for the MOH patients [31, 35, 36]. According to patient compliance, different withdrawal approaches were applied in MOH patients, but abrupt withdrawal was demonstrated to be more effective than tapered withdrawal based on a randomized study [37]. Similarly, our study found that abrupt withdrawal independently affected the headache outcome, which showed that abrupt withdrawal might be an appropriate approach for MOH patients to get an effective headache relief during the pandemic. Hence, available access to regular prophylactic medications and following abrupt withdrawal were likely to be important factors influencing the management of MOH patients during the pandemic. We could be able to improve headache symptoms of MOH patients by adjusting for these factors. High educational level was also significantly correlated to decreased headache intensity in this study. MOH patients with a higher educational level tended to be more willing to use prophylactic medications or consult neurologists than those with a lower educational level [38]. The higher educational level patients had, the more likely they were to obtain and follow education programs and guidelines of MOH managements, and thus to decrease headache intensity.

Headache specialists should pay particular attention to MOH patients with low educational level during the pandemic. They could make use of the telephone or telemedicine to conduct follow-up for MOH patients and maintain continuity of withdrawal and prophylactic medications through delivering medications to MOH patients’ home when they collaborated with commercial courier services.

As for days with acute medications per month, the majority of MOH patients reported unchanged or increased days with acute medication per month while fewer patients reported decreased days during the pandemic. Moreover, more than 70% of the original overusers still overused acute medications. In non-COVID-19 time, more than 60% of MOH patients were overuse free when treated by withdrawal combined with prophylactic medications in a multicenter and multinational study [35]. A possible explanation for this difference might be that MOH patients altered their drug administration passively or positively on their own. During the COVID-19 pandemic, regular prophylactic medications were difficult to access for MOH patients because of the inconveniences on transportation, seeking medical advice and so on, while acute medications were easy to get due to their ready accessibility (without prescription) and bargain price (average CNY 0.055 per tablet) as reported in China [32]. In our study, we found only 43.6% of MOH patients were able to follow the abrupt withdrawal and others were not able to. Although the tapered withdrawal could relieve withdrawal symptoms and improve patient acceptance by allowing restricted intake of acute medication during withdrawal [35, 39], patients with this tapered withdrawal approach were more psychologically dependent on headache acute medications than those with abrupt withdrawal at follow-up, and they might be less likely to complete the withdrawal [40]. Hence, some MOH patients with frequent headache attacks might be less willing to follow the abrupt withdrawal approach as they did in non-COVID-19 time [10] and still remained medication overuse especially when they did not receive regular prophylactic medications during the pandemic. Therefore, we should pay more attention to MOH patients’ health education and work to provide appropriate consultation for them if they followed the tapered withdrawal approach [31], by which we strived to help them effectively reduce the intake of acute medication during the pandemic.

We found male sex was significantly associated with decreased days with acute medication per month during the pandemic. Previously, no evidence was provided to support the positive correlation between male sex and acute medication administration. In our study, the cause of the sex differences in acute medication administration might come down to the following aspect. We found that psychological distress was lower in male sex than in female sex although it was not statistically significant. Compared with female sex, male sex was shown to be more inclined to have high resilience, a conception to reflect the good adaptation in the face of adversity, trauma, or even significant sources of stress [41]. This kind of high resilience in male sex might decrease the risk of medication overuse by relieving the psychological distress themselves during the pandemic [42–45]. Closer monitor on female patients was warranted about their acute medication intake per month in the therapeutic activity.

Furthermore, for psychological evaluation, none of the MOH participants in our study was found to suffer severe psychological distress indicated by K-6 scale during the COVID-19 pandemic surprisingly. Several reasons might account for this result. At first, hardly any recruited patients had epidemiological area exposure history and had COVID-19 cases in their community. Most of them were relatively unconcerned about the pandemic and spent little time following news of pandemic through social media. However, longer time of social media exposure was demonstrated be associated with the increased risk of psychological distress [46]. This could be the reason why the participants did not get distressed. Next, the resilience could be an important inner strength to weaken psychological distress during the pandemic [43, 44]. Riehm et al. found that middle-aged and older (≥50 years) adults tended more to report high resilience during the pandemic [45]. In our study, the mean age of the MOH participants was 51.6 (±10.5) year-old. We had reason to believe that most people in this group may be highly resilient to this the changes caused by COVID-19, showing a status with only mild psychological distress. Furthermore, although K-6 scale is a widely used scale for non-specific psychological distress, not all the specific symptoms of psychological distress could be screened out. Some more specialized scales, such as Hamilton Depression Rating Scale, may be further needed to assess emotional symptoms in MOH patients if it is possible during the pandemic.

Several limitations should be noted in our study. Firstly, this study was based on a relatively small sample of 78 MOH patients from a single center, which may be difficult to recruit sufficient subjects to provide convincing results as multiple centers. Whereas, the prevalence of MOH in the general population is 1% to 2% [9] and MOH is a secondary disease which may be neglected by many physicians who do not specialize in headache. Timely follow-up for the drug use and standard management of MOH in many other medical centers are lacking. Multi-center study with large sample on MOH patients will be further conducted if MOH could be better concerned and the management could be standardized under the regular epidemic prevention and control of COVID-19. On the other hand, West China Hospital, the core provider for medical sources in southwestern China, is a large tertiary medical center with abundant and representative sample of patients and we have a professional headache team to recognize those MOH patients. The participants in our study were still a representative group of MOH and the results were definitely reliable. Secondly, some data, such as psychological distress and COVID-19 related stressor, were difficult to collect and were dependent on self-reports. The information may vary with the status, educational level and personality of the individual who response to it. These might result in risk of bias. Considering these, we chose the classic published scales to design the questionnaire and items in the questionnaire were relatively typical and representative. Finally, a considerable number of patients could not complete the questionnaire online on the electronic device, so in this study we uniformly used telephone interview to complete the survey. It might also lead to the risk of bias for information collection, such as some self-rating scale (eg. K-6). We will continuously observe the psychosocial status and outcome of MOH patients with specialized scales and long-term follow-up to provide more clinical evidence in guiding peri-MOH management.

## Conclusions

Our findings reflect that MOH patients experienced a worse relief of headache symptoms and drug withdrawal during the pandemic. Available access to regular prophylactic medications was the significant independent factor for improvement of headache symptoms. Following abrupt withdrawal and high educational level were both significant factors for decreased headache intensity. Male sex was significantly associated with decreased days with acute medication. Headache specialists should give more awareness to education and counselling for MOH patients in the long-term fight against COVID-19 pandemic.

## Data Availability

Not applicable. The datasets of this current study are not available to public. Anonymous data can be obtained from the corresponding author upon appropriate request.

## Abbreviations

COVID-19: Coronavirus disease 2019
SARS-CoV-2: Severe acute respiratory syndrome coronavirus 2
MOH: Medication overuse headache
ICHD-III: The International Classification of Headache Disorders, 3rd edition
TTH: Tension-type headache
MIDAS: Migraine Disability Assessment Scale
K-6: Kessler 6-item
SPSS: Statistical Product and Service Solutions
